# On the Move: Correlation of Impaired Mobility with Spatial Navigation Ability in Persons with Multiple Sclerosis

**DOI:** 10.3390/brainsci14030277

**Published:** 2024-03-14

**Authors:** Alexis N. Chargo, Taylor N. Takla, Nora E. Fritz, Ana M. Daugherty

**Affiliations:** 1 Department of Psychology, Wayne State University, Detroit, MI 48202, USA; ana.daugherty@wayne.edu; 2 Institute of Gerontology, Wayne State University, Detroit, MI 48202, USA; 3 Neuroimaging and Neurorehabilitation Laboratory, Wayne State University, Detroit, MI 48201, USA; taylortakla@wayne.edu (T.N.T.); nora.fritz@wayne.edu (N.E.F.); 4 Translational Neuroscience Program, Wayne State University, Detroit, MI 48201, USA; 5 Department of Health Care Sciences, Wayne State University, Detroit, MI 48201, USA; 6 Department of Neurology, Wayne State University, Detroit, MI 48201, USA

**Keywords:** memory, walking, relapsing-remitting multiple sclerosis, Morris water maze

## Abstract

Spatial navigation ability is essential for independent living, and it relies on complex cognitive and motor processes that are vulnerable to decline in persons with multiple sclerosis (pwMS). The role of mobility in the physical act of navigation has been well documented; however, its association with cognitive processing that supports efficient navigation and recall of the environment is unknown. This study examined the relation between clinical mobility function and spatial navigation ability in pwMS. In a clinical sample of 43 individuals with relapsing-remitting MS (*M*_PDDS_ = 2; age 25–67 years), we assessed spatial navigation ability in a virtual Morris water maze that allowed for active search by controlling a joystick while seated at a computer, and subsequent free recall of environment details. Individuals with worse mobility (measured by slower forward and backward walking) traveled less efficient virtual navigation routes to the goal location and recalled fewer accurate details of the environment. A stratified analysis by disability revealed moderate–strong correlations for those with a low level of disability, and effects were attenuated in individuals with a high level of disability. Given that the virtual navigation task was performed while seated, evidence of any correlation with mobility suggests differences in navigation ability that cannot be ascribed to general walking impairment, and instead suggests a role for mobility impairment to modify cognitive processing supporting navigation in pwMS.

## 1. Introduction

Multiple sclerosis (MS) is a progressive, demyelinating disease of the central nervous system that affects millions of individuals around the world [1]. As the prevalence of MS continues to increase, it raises significant health concerns given the multifaceted nature of how the disease presents. Persons with multiple sclerosis (pwMS) are often challenged with both motor and cognitive deficits, with decline in cognition recognized as a prominent and disabling feature of the disease [2]. Common cognitive deficits in the disease include a decline in verbal [3] and visuospatial working memory [4], slowed information processing speed [5,6], and impaired executive functioning [7]. Together, these deficits can lead to difficulties in completing essential activities of daily living, reduced community-based participation, and ultimately, a diminished quality of life. While much of the prior literature has focused on exploring various aspects of cognitive impairment in pwMS, one application that remains relatively uncharted is spatial navigation—a critical, though often overlooked, everyday ability that is essential for independent living. 

Spatial navigation is a behavior that is composed of cognitive wayfinding ability and movement towards a specific goal location [8]. Wayfinding encompasses a wide range of cognitive functions that allow for an individual to orient within the environment and plan navigation routes [9]. Wayfinding is thought to reference a cognitive map—a mental representation of the environment that is created through experience and stored in memory [10]—which supports route planning and adaptation in order to reach a desired goal destination [11]. 

The study of spatial navigation ability considers the efficiency of the traveled route to the goal location [12], in addition to memory of environment details [13,14]. The traveled distance or time between the start and goal locations are common measures of navigation ability, for which greater efficiency strongly correlates with better working memory function [15]. In comparison, the ability to recall details about the environment is strongly associated with episodic memory function [16,17]. Efficient travel routes (relatively short distance and time) often correlate with the ability to recall more details in the environment, presumably due to reference of a cognitive map in memory to plan the travel route. Nonetheless, measures of navigation efficiency and memory of the environment show differential vulnerability in aging and other clinical populations, as well as distinct neural correlates [18,19]. Their mutual study, therefore, can provide different insights into the source of decline in navigation ability. These different cognitive functions that support spatial navigation ability are affected in pwMS; therefore, it is plausible that these individuals would experience deficits in navigation ability that may contribute to compromised daily living. 

**Figure 1:**
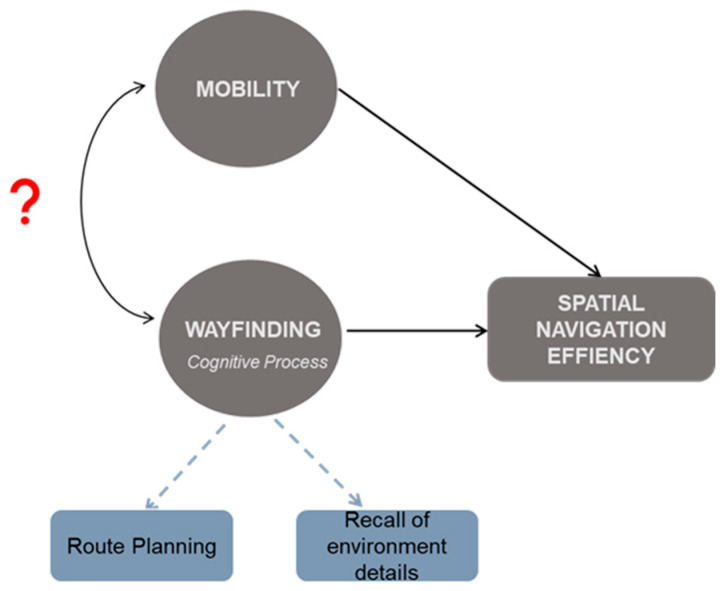
Conceptual diagram illustrating the hypothesized association of mobility with spatial navigation ability and the related wayfinding cognitive processing, beyond merely locomotion. Curved double-headed arrow with the red question mark represents what is unknown; solid arrows are indicative of what has been established in the literature in healthy aging and related clinical populations, but little has been studied in pwMS.

One of the relative unknowns, however, is the role of mobility impairment in navigation ability beyond the act of movement. MS-specific deficits in mobility, including slowed gait speed and poor postural control [20,21,22], are certain to impact efficient navigation when walking (i.e., greater time during route travel). Difficulty with more complex mobility, such as backward walking (BW) or side-stepping, may also impact efficient navigation when walking. Our lab has previously shown that working memory and visuospatial memory contribute to the relation between disease severity and BW velocity in pwMS [23] and that BW better predicts falls in pwMS than forward walking (FW) [24]. However, beyond locomotion, mobility and its potential to support cognitive processing that is necessary for successful navigation may also be impaired (See Figure 1). 

Despite the rich research literature, studies of spatial navigation ability in pwMS are rare. A single study reported impairment in general wayfinding ability [25]; however, the assessment was administered online using passive viewing of an environment, and, therefore, there are no data concerning active navigation routes. Importantly, real-world navigation includes autonomous route planning and travel; thus, impairment in autonomous navigation has the greatest consequence to independent living. We can begin to isolate the association of mobility with cognitive components of autonomous spatial navigation apart from motor actions using virtual environments, where pwMS control navigation while seated at a computer. Virtual navigation tasks within laboratories have been well-validated against real-world navigation [26] and offer study control of potential confounds when working with pwMS. 

Therefore, the purpose of this study was to evaluate associations of mobility, specifically FW and BW, to navigation efficiency (virtual distance and time), and free recall of environment details following the completion of a virtual navigation paradigm. The MS patient population is ideal for understanding the complex cognitive–motor interaction in human spatial navigation ability, and the results can meaningfully inform interventions for pwMS to maintain independence. The reported results are an initial step in understanding a role of mobility in supporting the cognitive processing and recall of environment details that are essential for successful real-world spatial navigation. 

## 2. Materials and Methods

### 2.1. Participants

A convenience sample of 43 individuals (age 25–67 years; *M* = 48.16, *SD* = 10.28) with relapsing-remitting multiple sclerosis (RRMS) were recruited from the Metro Detroit area as part of a cross-sectional study. All study procedures were approved by the Wayne State University Institutional Review Board, and participants provided written informed consent prior to engaging in data collection procedures.

Selection criteria required participants to be at least 18 years of age at enrollment. All participants must have denoted a Patient-Determined Disease Steps (PDDS) score of ≤6, indicating that they could ambulate with or without an assistive device ≥50% of the time. Participants were excluded from participation if they reported an MS relapse or exacerbation within 30 days of testing, were diagnosed with a neurological condition other than MS, had any acute orthopedic injuries or other major conditions that would impede cognitive and motor function, and were unable to comprehend and follow study-related commands. 

### 2.2. Testing Procedures

Participants completed all testing on a single assessment occasion. All participants completed demographic questionnaires, as well as the PDDS [27] to determine disease severity. Participants also completed a series of mobility assessments (i.e., timed walking tests), as well as neuropsychological testing which included assessment of navigation abilities. 

#### 2.2.1. Spatial Navigation Ability Assessment

Spatial navigation was assessed using a virtual adaptation of the traditional Morris water maze [12,15]. The virtual Morris water maze (vMWM) assessment was administered on a computer, where participants remained seated throughout the assessment. The virtual environment was viewed from a first-person perspective. Participants were instructed to move through the environment by controlling a joystick with their right hand. Participants traveled at a constant speed during exploration and could stop as desired; movement in the backward direction was prohibited. 

*Practice.* Before testing, participants were first exposed to a practice pool environment to familiarize themselves with movement through the environment. The practice pool environment consisted of five visible platforms labeled A–E; participants were instructed to cross the visible platforms in alphabetical order, with successful completion of the trial confirming satisfactory control of the joystick. 

**Figure 2:**
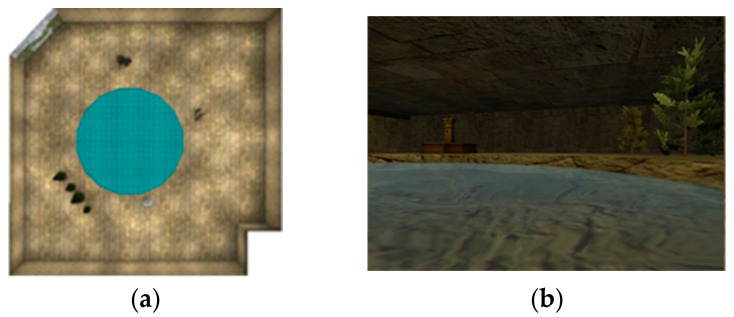
Overhead view of the virtual Morris water maze environment (**a**). The circular pool is illustrated in blue in the center of the room. First-person perspective (**b**) showing 2 of the object cues that surround the pool.

*Virtual Morris Water Maze (vMWM)*. The on-screen virtual environment consisted of a circular pool, placed within a larger room. There were several distinct objects, or cues, located around the perimeter of the pool, as well as 2 unique wall features, which could be used to help guide navigation (See Figure 2 for depiction of vMWM environment). A goal platform was hidden beneath the surface of the pool, and participants were instructed to navigate to the platform as fast as possible. Because the platform was hidden, the participant must use the available cues in order to locate its position within the virtual environment. Trials were terminated at the participant’s first intersection with the platform, where movement would cease, the platform would raise above the surface of the water, and a sound would be made. Trials were fixed to 2 min and terminated if the participant did not successfully locate the platform within that time. Navigation efficiency data were analyzed only for trials that were successful.

The goal platform was centered in one pool quadrant and remained in the same location throughout all 5 navigation learning trials. Participants began each trial from a different location situated within one of the 3 quadrants that did not contain the goal platform. All starting locations were positioned at an equal distance from the platform, with facing direction at each starting location randomized per trial. Navigation efficiency was measured as distance (virtual units) and time (s) traveled from the starting location to the first intersection of the hidden platform. Following completion of the learning trials, participants completed a fixed 1-minute probe trial; however, this data was not included in the reported analyses. 

*Recall of Environment Details*. Following the vMWM learning trials, participants completed a map replication task to assess free recall of environment details. Participants were provided a blank piece of paper and instructed to draw an overhead view of the virtual environment, including as many details as possible, and marking the location of the hidden platform with an “X” [28]. Correct free recall was scored based on the number of cues (i.e., objects and wall features) included in the map replication, as well as their correct location relative to the platform. A higher score indicates better memory for environment details. 

#### 2.2.2. Mobility Assessments

*Timed 25-Foot Walk (T25-FW)*. Participants completed the T25-FW in the forward (FW) and backward (BW) direction at a self-selected comfortable and fast pace over a distance of 25 feet. The T25-FW has been determined as a valid and reliable assessment of mobility in pwMS [29,30,31]. 

For both FW and BW, participants completed a total of four trials—two trials at their self-selected comfortable pace, and two at their fast pace where they were instructed to walk as quickly as possible while maintaining safety. Time to complete each trial was recorded in seconds, and averages for comfortable and fast-paced time were computed for FW and BW. For all trials, participants wore a gait belt and were accompanied by a member of the research team to ensure safety. 

### 2.3. Statistical Analyses

Prior to running analyses, data screening procedures were conducted using SPSS V 28. A large portion of the data presented with non-normal, skewed univariate distributions (*z* > |3.1|). Seven univariate outliers were detected (*z* > |3.29|), and four individual cases were identified as multivariate outliers (Mahalanobis distance, critical χ^2^ (*df =* 3) = 16.266, α = 0.001). Given these data attributes, non-parametric statistical tests were selected. Spearman rho correlations were computed to examine the relation between clinical mobility (comfortable and fast FW, BW) and measures of spatial navigation, including navigation efficiency (distance, time) and cognitive map recall. Significance testing for all analyses was evaluated at α = 0.05. Our sample of 43 pwMS will provide 80% power to detect moderate to large effects of significance (*d* = 0.41, α = 0.05). Results were replicated in analyses with comparable effects when outliers were removed, thus demonstrating negligible bias in interpretation. Therefore, findings are reported and interpreted using the entire sample. 

*Consideration of Potential Covariates for Analysis.* Despite the literature highlighting a general age-related deficit in navigation [12,32], our sample had very weak and non-significant correlations of age with navigation efficiency measures of time (*ρ* = 0.211, *p* = 0.174) and distance (*ρ* = 0.161, *p* = 0.301), as well as recall of environment details (*ρ* = −0.224, *p* = 0.154); therefore, age was not included as a covariate for analysis. Furthermore, because our sample was predominantly female, which is consistent with the prevalence of MS [33] in the general population, there was insufficient representation to include sex as an additional covariate. A weak, non-significant association of PDDS with navigation measures suggested against including the variable as a covariate. However, to evaluate a potential interaction with disease severity, descriptive and correlation analyses were estimated and stratified by median PDDS: high as compared to low disability levels with a clinical cut-off of PDDS > 3.

## 3. Results

A clinical sample of 43 (81.4% female) participants with RRMS were recruited for participation. Participants had an average symptom duration of 17.59 years (*SD* = 9.98). See Table 1 for a complete report of the sample demographics.

### 3.1. Poor Mobility Was Associated with Worse Virtual Navigation Ability in pwMS

**Figure 3:**
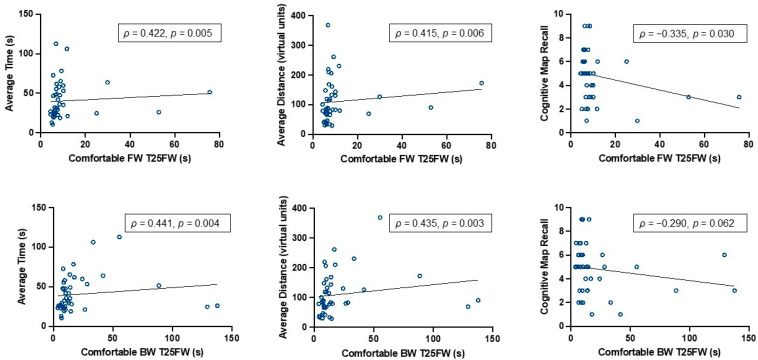
Bivariate relations between comfortable FW and BW with measures of spatial navigation ability. Note. Spearman rho (*ρ*) correlation values for FW and BW with spatial navigation efficiency measures (average virtual distance and time traveled to reach goal platform location), in which higher values indicate worse performance. Memory of the environment was assessed with the number of accurately recalled environment details, in which lower values indicate worse performance.

Poor mobility, as indicated by slower T25FW walking performance, was associated with inefficient virtual route travel and worse recall of environment details. PwMS who had a slower, more comfortable FW time traveled a greater virtual distance (*ρ* = 0.415, *p* = 0.006), took longer to reach the goal platform (*ρ* = 0.422, *p* = 0.005), and recalled fewer accurate environment details (*ρ* = −0.335, *p* = 0.030) (See Figure 3). A similar trend was observed for pwMS with a slower, more comfortable BW time (Table 2), with the exception of the recall of environment details, as there was no significant association between these measures (*ρ* = −0.290, *p* = 0.062). Interestingly, fast-paced FW was not associated with any of the navigation measures. However, slower fast-paced BW time was associated with a longer time spent traveling to reach the goal platform location (*ρ* = 0.364, *p* = 0.018). See Table 2 for all Spearman correlation values.

### 3.2. PDDS-Stratified Correlations Reveal Similar Associations between Mobility and Navigation Performance for pwMS Who Have Lower Disability Levels

To consider the potential for differential associations among mobility and spatial navigation ability as a function of disease severity, descriptive analysis was repeated with the sample stratified by high (≥3) and low (<3) PDDS scores with a score of 3 indicating a moderate disability level where gait dysfunction begins to emerge [27]. See Table 3 for a demographic profile of each group.

The pattern of results observed in the whole sample was largely replicated in each of the stratified disability groups, which suggests that the relation between mobility and spatial navigation ability lies on a linear continuum across disease severity. In the low-level disability group, slower, comfortable FW was associated with a greater virtual distance (*ρ* = 0.392, *p* = 0.039) and time (*ρ* = 0.587, *p* = 0.001) traveled to reach the goal platform, and worse recall of environment details (*ρ* = −0.418, *p* = 0.027). Similarly, pwMS with low-level disability with slower, comfortable BW traveled less efficient routes to the goal platform (distance: *ρ* = 0.412, *p* = 0.030; time: *ρ* = 0.571, *p* = 0.001) and recalled fewer details about the virtual environment (*ρ* = −0.500, *p* = 0.007). There were no significant associations between fast FW and measures of navigation. Conversely, there was a strong correlation between fast-paced BW, average time traveled (*ρ* = 0.506, *p* = 0.006), and recall of environment details (*ρ* = −0.501, *p* = 0.007). Within the high-level disability group, many of the effects had comparable effect sizes; however, the associations with free map recall were notably attenuated (See Table 4).

## 4. Discussion

The present study aimed to examine the associations between FW, BW, and measures of spatial navigation ability in pwMS. In general, pwMS who had slower comfortable FW times had worse virtual navigation efficiency, and poor recall of environment details. Comfortable BW was associated only with distance and time navigation efficiency measures, and not free map recall. Furthermore, we found no significant associations of the spatial navigation assessment with fast FW or BW, with the exception of fast BW and travel time. Considering potential differences by disease severity, a similar pattern of effects as the whole sample were observed in those with lower disability levels, and some associations in the high-level disability group were attenuated. Taken together, we provide compelling initial evidence of mobility impairment modifying complex route planning and memory processes that can account for impaired navigation ability in pwMS.

Studies of MS disease effects on spatial navigation ability are rare. At the time of this writing, there are two other studies with an MS-specific clinical sample [25,34], which suggested impairment in cognitive processes associated with navigation, such as route knowledge [25,34] and landmark-based allocentric navigation [25,34] in pwMS as compared to healthy controls. We add to this literature with additional evidence of impairment on a continuum with disease severity within a group of pwMS, and by isolating cognitive components of active spatial navigation that correlated with independent assessment of motor function. This work has important implications to strengthen our understanding of how cognition and mobility interact to support real-world navigation and improve independent living outcomes for pwMS.

Our research demonstrates a dependency of spatial navigation ability on mobility that is at least in part due to cognitive differences, which includes measures of navigation efficiency (distance, time). The association of mobility impairment and slowed walking during navigation is of course expected; however, because the navigation task we report was in a virtual environment explored by a joystick while the participant was seated, the correlations we report between navigation efficiency and mobility suggest an impairment in cognitive processing instead of physical movement. Based on the extant literature, mobility is a construct composed of physical movement (i.e., locomotion) and the cognitive component of motor planning [35,36,37]. There is a significant amount of overlap in motor planning abilities with cognitive components of spatial navigation ability, including efficient route planning that supports successful navigation, and mutual correlations with working memory and executive functions [38,39,40]. Another study comparing pwMS to healthy controls who were freely walking in a real-space analog of the MWM identified a similar association of the disease with a greater navigation distance error [25,34]. Poor efficiency could reflect impaired motor planning, as well as a decline in working memory, and visuospatial learning—all of which are often impaired in pwMS [40,41,42]. Compared to FW, BW presumably has greater cognitive and motor demands, and correlates with visuospatial working memory [23,43,44,45]; therefore, the trend for slightly stronger correlations of BW with efficiency measures as compared to the map recall task is interesting. Given the shared overlap in cognitive architecture, mobility impairment may modify visuospatial working memory and motor planning components that support navigation beyond merely locomotion. Future studies with longitudinal measurement are necessary to determine if the association of mobility and navigation efficiency we observe is due to this shared set of cognitive correlates, or if mobility impairment may precede decline in spatial navigation ability.

In the stratified analysis, findings confirm that the association of mobility and spatial navigation ability are present across the continuum of disability, and most strongly evident in pwMS who have low to moderate clinical impairment. Although several of the correlations were of comparable effect size in the group with a high level of disability, some were attenuated. One interpretation for this is a general loss of complex cognitive–motor function with a high level of disability and the spatial navigation task may become less sensitive to detect individual variability within that group. Nonetheless, the pattern of associations we observed within this sample is consistent with the vulnerability of cognitive processing supporting spatial navigation ability during the progression of mobility impairment in MS. This further motivates the need for earlier intervention and the potential application of spatial navigation assessment as an end target that could be responsive to change in pwMS at early stages of disability before irreversible cognitive decline has taken hold.

The association between mobility and the recall of environment details in the whole sample, as well as in the stratified analysis, is particularly thought-provoking. Unlike navigation efficiency measures that are often strongly associated with working memory and procedural skill that have obvious parallels to motor function [15], the recollection of specific environment details and their spatial location rely heavily on declarative memory function. A common understanding of mobility in spatial navigation emphasizes locomotion as the outcome of a complex cognitive process, but there has been little consideration for mobility to modify the underlying memory processes that may guide navigation decisions. In the available studies of navigation in pwMS, individuals tend to have impaired memory of landmarks [25] and poor allocentric landmark knowledge [34]—both of which are essential for the creation, maintenance, and use of a map of the environment that is stored in memory to support navigation. While mobility was not formally evaluated in either of these prior studies, it was even suggested that impaired mobility can affect the acquisition of landmark knowledge [25], most likely by limiting exploration and exposure to landmarks that are necessary for creating a mental representation of the environment [26]. We were able to more directly investigate this as participants could freely move without limitation in the virtual environment, and so any association of mobility with recall of the environmental landmarks most likely reflects differences in memory processes or top-down navigation strategies. This implies a role for mobility to alter antecedent cognitive processing that supports spatial navigation, independent of the physical act of locomotion, as illustrated in Figure 1. Future research should consider the potential overlap between cognitive motor planning and the wayfinding process in these traditional navigation tasks. For pwMS, this highlights the possibility of additional intervention routes that leverage memory processing and recall strategies to bolster independent spatial navigation ability, and, in turn, improve the quality of daily life.

### Limitations

While this research provides novel, valuable insights about spatial navigation in pwMS, it is important to acknowledge certain limitations that might impact the interpretation of our findings. Given the small sample size of 43 pwMS, and because our sample consists of primarily females, the generalizability of our findings may be limited in the general MS population. However, MS exhibits a disproportionate impact on women, with a threefold higher prevalence in women compared to men [46]. Therefore, our sample adequately reflects this clinical population. Further, we acknowledge that the average level of disability in our sample was within a modest range (average PDDS = 2). Despite this limitation, our findings from a sample with a relatively low level of disability provide valuable insights into the early stages of MS when interventions may have the greatest impact. Importantly, our study was the first to explore the effects of mobility impairments on cognitive processes supporting navigation abilities; however, we only considered time to complete the T25FW as a measure of mobility function. Future studies should consider other measures of mobility, including dynamic gait performance and postural control. It would also be of benefit for future studies to inquire about fall histories and fear of falling, as these can have a negative impact on one’s navigation abilities (i.e., avoiding obstacles or only traveling certain routes due to fear of falling, leading to limited exploration of the environment). Additionally, while the vMWM is a well-validated task commonly used in laboratory assessment of navigation, it has weak ecological validity. Nonetheless, through the use of the vMWM, we are able to minimize the direct effect of gait impairments on wayfinding ability, which allows for a strong measure of the impact of mobility function on cognitive processing that supports navigation.

## 5. Conclusions

The reported findings begin to provide a foundation for understanding the complex interplay between cognition and mobility using navigation as a specific real-world application. We demonstrated that there is in fact a relation between mobility and wayfinding, or the cognitive processing that supports successful navigation, in a seated virtual navigation task. Because the reported associations were evident across a continuum of disability levels, it suggests that there is a potential clinical use for incorporating assessments of spatial navigation ability as a sensitive measure for detecting early cognitive decline in a clinical sample with a low level of disability. Earlier detection of complex cognitive–mobility decline would provide a window of opportunity for interventions to have the greatest potential to mitigate further cognitive loss. The associations further suggest that cognitive interventions that target wayfinding ability may be one way to promote the maintenance of complex cognitive–mobility function for longer as MS pathology progresses. This collectively would align with the goal of maintaining and improving navigation abilities in those who are most vulnerable to decline, ultimately facilitating independence and improving their overall quality of life.

## Figures and Tables

**Table 1:** Description of sample demographics.

Variable	Descriptive Statistic
Sample Size	43
Female, *n* (%)	35 (81.4%)
Age (*M* ± *SD*, years)	48.16 ± 10.28
Symptom Duration (*M* ± *SD*, years)	17.59 ± 9.98
Disease Severity (*M* ± *SD*, PDDS)	2.00 ± 1.98

Note. Demographic profile is reported for the recruited clinical sample of persons with RRMS that was included for analysis. Sample means and standard deviations are reported (*M* ± *SD*).

**Table 2:** Bivariate correlations among mobility and navigation measures for the entire sample.

Measure	1	2	3	4	5	6	7
1. Comfortable FW	--						
2. Comfortable BW	0.834 **	--					
3. Fast FW	0.787 **	0.663 **	--				
4. Fast BW	0.779 **	0.965 **	0.689 **	--			
5. Average Distance	0.415 **	0.435 **	0.137	0.284	--		
6. Average Time	0.422 **	0.441 **	0.221	0.364 *	0.793 **	--	
7. Map Free Recall	−0.335 *	−0.29	−0.159	−0.195	−0.516 **	−0.440 **	--

Note. Spearman rho (*ρ*) correlations are reported for the bivariate relation between comfortable and fast-paced forward (FW) and backward (BW) times, and spatial navigation ability indexed by average travel distance and time in the virtual environment, and free recall of environment details. * p < 0.05, ** p < 0.01, unadjusted.

**Table 3:** Sample demographics stratified by PDDS.

	Descriptive Statistic	
Variable	Low Disability	High Disability
Sample Size	28	15
Female, *n* (%)	24 (85.7%)	11 (73.3%)
Age (*M* ± *SD*, years)	46.96 ± 9.89	50.40 ± 10.96
Symptom Duration (*M* ± *SD*, years)	17.42 ± 10.11	17.87 ± 10.08
Disease Severity (*M* ± *SD*, PDDS)	0.75 ± 0.80	4.33 ± 1.23

Note. Demographic profile is reported for the clinical sample of persons with RRMS stratified into low-level and high-level disability groups. Sample means and standard deviations are reported (*M* ± *SD*).

**Table 4:** Bivariate correlations between mobility and navigation measures when stratified by PDDS.

	Low Disability (n = 28)							High Disability (n = 15)						
Measure	1	2	3	4	5	6	7	1	2	3	4	5	6	7
1. Comfortable FW	--							--						
2. Comfortable BW	0.853 **	--						0.721 **	--					
3. Fast FW	0.696 **	0.519 **	--					0.916 **	0.851 **	--				
4. Fast BW	0.826 **	0.945 **	0.564 **	--				0.675 **	1.00 **	0.851 **	--			
5. Average Distance	0.392 *	0.412 *	0.068	0.269	--			0.332	0.289	0.160	0.253	--		
6. Average Time	0.587 **	0.571 **	0.284	0.506 **	0.764 **	--		0.314	0.343	0.182	0.327	0.904 **	--	
7. Map Free Recall	−0.418 *	−0.500 **	−0.273	−0.501 **	−0.519 **	−0.489 **	--	−0.155	−0.141	−0.120	−0.184	−0.392	−0.379	--

Note. Spearman rho correlations describing the bivariate relation between comfortable and fast-paced forward (FW) and backward (BW) times, average distance, average time, and recall of environment details for low and high disability groups. * *p* < 0.05, ** *p* < 0.01, unadjusted.

## Data Availability

The data presented in this study are available on request from the corresponding author. The data are not publicly available due to data containing information that could compromise the privacy of research participants.
